# The Relationship Between Parental Smartphone Addiction and Child Problematic Media Use: Mediating Roles of Child–Parent Conflict and Parental Psychological Distress and Validation of the Child–Parent Relationship Scale‐Short Form in Arabic

**DOI:** 10.1002/puh2.70209

**Published:** 2026-04-01

**Authors:** Nicole Tannous, Marilyne Aouad, Abdallah Chahine, Rabih Hallit, Diana Malaeb, Sami El Khatib, Fouad Sakr, Mariam Dabbous, Souheil Hallit, Feten Fekih‐Romdhane, Sahar Obeid

**Affiliations:** ^1^ School of Medicine and Medical Sciences Holy Spirit University of Kaslik Jounieh Lebanon; ^2^ Department of Infectious Disease Bellevue Medical Center Mansourieh Lebanon; ^3^ Department of Infectious Disease Notre Dame des Secours University Hospital Byblos Lebanon; ^4^ College of Pharmacy Gulf Medical University Ajman United Arab Emirates; ^5^ Department of Biomedical Sciences, School of Arts and Sciences Lebanese International University Beirut Lebanon; ^6^ Center for Applied Mathematics and Bioinformatics (CAMB) Gulf University for Science and Technology (GUST) Hawally Kuwait; ^7^ School of Pharmacy Lebanese International University Beirut Lebanon; ^8^ Applied Science Research Center Applied Science Private University Amman Jordan; ^9^ Department of Psychiatry “Ibn Omrane” The Tunisian Center of Early Intervention in Psychosis, Razi Hospital Manouba Tunisia; ^10^ Faculty of Medicine of Tunis Tunis El Manar University Tunis Tunisia; ^11^ Department of Psychology and Education, School of Arts and Sciences Lebanese American University Byblos Lebanon

**Keywords:** anxiety, closeness, parental psychological distress, parental smartphone addiction, parent–child conflict, parent–child relationship, problematic media use

## Abstract

**Objective:**

To study how parental psychological distress and parent–child conflict (PCC) mediate the relationships between smartphone dependence in caregivers and their children's problematic usage of media and to validate the Child–Parent Relationship Scale‐Short Form (CPRS‐SF) in Arabic, a valuable tool that assesses intrafamilial connections.

**Methods:**

This cross‐sectional study enrolled 892 parents with kids aged between 6 and 10 years of age. The confirmatory factor analysis tested the two‐factor structure of the CPRS‐SF scale, and the mediation analyses explored the link between parental smartphone addiction (PSA) and child media use.

**Results:**

The Arabic version of the CPRS‐SF showed strong reliability for both the Conflict (*α* = 0.88) and Closeness (*α* = 0.91) subscales. PSA was directly and positively associated with child problematic media use and indirectly via two mediators: higher psychological distress in parents and greater PCC and their children. However, anxiety and closeness between parents and children were not significant mediators.

**Conclusion:**

Our findings indicate that children's media habits are associated with parental psychological well‐being and the quality of parent–child relationships. These results highlight the importance of considering family emotional and relational contexts when addressing child problematic media use and suggest that awareness of parental distress, PCC, and problematic smartphone use may be relevant for clinicians and educators working with families.

## 1 Introduction

Smartphones have become essential tools in modern society, as they facilitate communication, access to information, and the use of multiple applications within a single device [1]. In 2024, around 5.68 billion people worldwide used mobile phones, and 85% of these devices were smartphones [2]. Despite their widespread utility, growing concern has emerged regarding the potential for excessive and problematic smartphone use. Parental smartphone addiction (PSA) refers to excessive and compulsive smartphone use that interferes with parents’ daily responsibilities, attention, and caregiving behaviors [3, 4, 5, 6, 7].

### 1.1 The Link Between PSA and Child Problematic Media Use

PSA has been consistently associated with higher levels of child problematic media use [8]. According to the compensatory internet use theory, child problematic media use may emerge when children attempt to compensate for unmet emotional needs, cope with feelings of rejection and uncertainty, or regulate psychological distress [8]. When media becomes a primary source of comfort, children may gradually disengage from interpersonal interactions and develop increased dependence on screens [8]. Such patterns have been linked to adverse outcomes, including poorer social skills, reduced attention span, and lower academic performance [8]. Several theoretical frameworks help explain why PSA may be associated with elevated child problematic media use. The Social Learning Theory suggests that parents who model problematic media behaviors are less likely to establish or enforce effective media boundaries, partly because doing so would require self‐regulation on their own use [5, 9, 10]. Conversely, clear parental rules and consistent communication have been significantly associated with lower risks of compulsive internet use in children [10, 11]. Therefore, this modeling pattern illustrates how parental smartphone behaviors may be linked to children's media habits. Nevertheless, child problematic media use is widely acknowledged as a complex, multifactorial phenomenon rather than a direct consequence of PSA alone. The interactional theory of childhood problematic media use (IT‐CPU) helps explain that child problematic media use arises from interacting distal, proximal, and maintaining factors [12]. Proximal factors may include parental media habits and mental health, whereas maintaining factors include the quality of the parent–child relationship. On the basis of this theory, parental psychological distress and parent–child conflict (PCC) are conceptualized as not merely as correlates but as potential mechanisms linking PSA to child problematic media use.

### 1.2 Mediating Pathways Through Parental Mental Health and the Parent–Child Relationship

**FIGURE 1:**
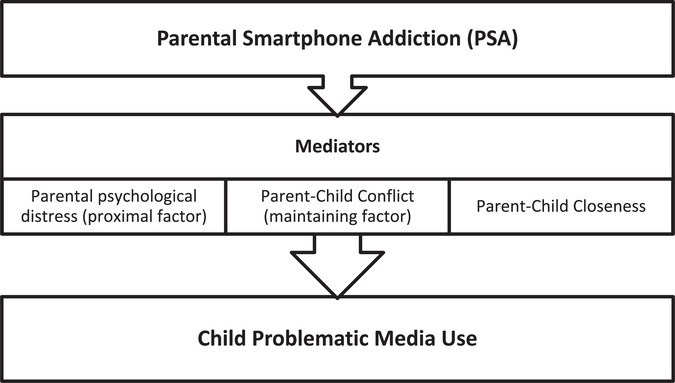
Conceptual framework of the hypothesized mediation model based on the interactional theory of childhood problematic media use [21].

Building on the IT‐CPU model, the present study proposes that the association between PSA and child problematic media use may be explained through two mediating pathways: parental mental health and the quality of the parent–child relationship (Figure 1). Parental psychological distress represents a key proximal factor influencing parenting behaviors [13]. Psychological distress can manifest as feelings of worthlessness and difficulty concentrating, which can compromise a parent's capacity for engaged and consistent caregiving [14, 15, 16]. Parents experiencing psychological distress may encounter greater difficulty monitoring children's screen use or enforcing recommended screen time guidelines, such as the 1‐h daily limit recommended by the American Academy of Pediatrics, thereby increasing children's susceptibility to child problematic media use [17, 18, 19, 20].

The parent–child relationship constitutes a second critical pathway potentially linking PSA to child problematic media use. Poor relationship quality, characterized by high levels of conflict and low parent–child closeness, has been associated with greater risk of child problematic media use [22]. Frequent smartphone engagement may render parents distracted, emotionally unavailable, or less responsive, which children may perceive as rejection or neglect [23]. Over time, such interactions may contribute to weakened attachment bonds and increased PCC [24, 25, 26]. In response to these relational deficits and unmet emotional needs, children may turn to media to fulfill their unmet needs for comfort [27]. Research showed that harsh parental criticism and reduced closeness are significant markers of problematic media use in early childhood [22]. In contrast, warm and supportive parent–child relationships are associated with healthier media practices, whereas strained relationships may foster a vicious cycle where both parents and children retreat to screens [7, 22, 28]. Accordingly, the quality of the parent–child relationship is central to children's media habits [29, 30].

A widely used tool for assessing parent–child relationship quality is the Child–Parent Relationship Scale‐Short Form (CPRS‐SF). This 15‐item scale assesses constructive and maladaptive parent–child interactions through two dimensions: conflict and closeness [31]. The CPRS‐SF was originally validated among white families in the United States and has since been examined across diverse cultural contexts, demonstrating generally acceptable factor structure and internal consistency [32, 33]. Notably, psychometric studies conducted in Turkey and the United Kingdom identified one item (Item 4) with consistently low factor loadings, leading to its removal [33, 34]. Despite its widespread use, the CPRS‐SF has not been validated among Arabic‐speaking families in Lebanon. Given the distinct stressors, family structures, and cultural norms in Lebanon, formal validation is necessary to ensure the scale's reliability, validity, and cultural appropriateness [34, 35].

#### 1.2.1 Rationale and Objectives

The specific pathways linking PSA with child problematic media use through parental psychological distress and PCC remain underexplored in the Lebanese context. Nearly 20% of Lebanese adults report smartphone addiction symptoms, a prevalence reported to be higher than that observed in several Western countries [5, 6]. Coupled with evidence that approximately 95% of Lebanese youth spend an average of 3 h per day on social media, these patterns underscore an urgent need to better understand family‐level mechanisms associated with media overuse in the Lebanese context [7, 36]. Accordingly, this study aimed to (1) examine a mediation model assessing the associations between PSA and child problematic media use through parental psychological distress and PCC and (2) adapt and evaluate the psychometric properties of the CPRS‐SF in an Arabic‐speaking Lebanese sample. Therefore, the contribution of this article is twofold. First, the study psychometrically validates the CPRS‐SF in Arabic and examines its measurement invariance across sex. Second, the mechanistic pathways linking PSA to child problematic media use are investigated for the first time in a Lebanese context and with covariate adjustment. On the basis of the reviewed literature and the IT‐CPU framework, we hypothesized that (H1) PSA would be positively and directly associated with child problematic media use; (H2) parental psychological distress and PCC would mediate the association between PSA and child problematic media use; and (H3) the Arabic version of the CPRS‐SF would demonstrate good validity and reliability.

## 2 Methods

### 2.1 Participants

We conducted a cross‐sectional study among a sample of Lebanese parents in August 2024. Data were collected using an online questionnaire administered in Arabic and distributed via a snowball sampling technique. Eligible participants were Lebanese parents aged ≥18 years who had at least one child aged 6–10 years. The exclusion criteria were being non‐Lebanese, being younger than 18 years, declining participation, or not having children aged 6–10 years. All participants gave us their informed consent before participating. A total of 892 individuals filled the survey with a mean age of 37.22 years.

#### 2.1.1 Instruments

The questionnaire was written in Arabic. The first page asked about sociodemographic information such as age, gender, marital status, kids, education level, occupation, screen time per day, the household crowding index, and the number of daily working hours. Note that the household crowding index equals the total number of people in the household divided by the number of rooms, excluding the kitchen and bathrooms [37].

The following pages contained multiple scales.

#### 2.1.2 Problematic Media Use Measure (PMUM)

The PMUM is a parent‐report questionnaire validated in Arabic that was developed to assess screen media addiction in children aged 4–11 years [38]. The short form, composed of nine items, was used. Items are rated on a five‐point Likert scale (1 = never and 5 = always), yielding scores from 9 to 45, with higher scores indicating more problematic media use. The PMUM has demonstrated excellent internal consistency (Cronbach's *α* = 0.97) and good convergent validity with measures of child psychosocial functioning [26]. The Arabic version also showed strong reliability (Cronbach's *α* = 0.90). In the current study, Cronbach's *α* was 0.93.

#### 2.1.3 Patient Health Questionnaire‐4 (PHQ‐4)

The PHQ‐4 is an ultra‐brief screening instrument developed by [39] to assess symptoms of psychological distress. It consists of four items rated on a four‐point Likert scale (0 = not at all to 3 = nearly every day), assessing the frequency of depression (two items) and anxiety (two items) symptoms over the previous 2 weeks. Total scores range from 0 to 12, with higher scores indicating more psychological distress. The PHQ‐4 was originally designed to provide a global measure of psychological distress and has been widely used in epidemiological and public health research for this purpose [39]. In the present study, the PHQ‐4 total score was used as an indicator of overall psychological distress in mediation analyses. This approach was chosen because each subscale (anxiety and depression) comprises only two items, which limits their standalone psychometric robustness. In addition, the anxiety and depression subscales showed very high correlations with the total PHQ‐4 score, indicating substantial overlap. The use of the total score therefore provides a more reliable and parsimonious representation of overall psychological distress. For descriptive purposes, internal consistency was acceptable for the total psychological distress score (Cronbach's *α* = 0.85) in the present sample. The scale was validated in Arabic, showing a Cronbach's *α* of 0.86 [40].

#### 2.1.4 Smartphone Addiction Scale (SAS)

The SAS was originally developed by [41] to assess the level of PSA. The original versions consist of 33 items [42]. Items are rated on a six‐point Likert scale (1 = strongly disagree to 6 = strongly agree). The SAS has shown excellent internal consistency (Cronbach's *α* = 0.967) and strong convergent validity [42]. The Arabic‐validated SAS short version contains 10 items, with total scores ranging from 10 to 60 [43]. The SAS‐SV demonstrated excellent reliability in the current study (Cronbach's *α* = 0.93).

#### 2.1.5 Child–Parent Relationship Scale‐Short Form

The CPRS was developed by to assess parents’ perceptions of their relationship with their child, focusing on conflict, closeness, and dependency. The original scale includes 30 items rated on a five‐point Likert scale (1 = definitely does not apply to 5 = definitely applies) [44, 45]. There also exists a short form, containing 15 items. Seven items are for conflict (CPRS1, CPRS3, CPRS5, CPRS6, CPRS7, CPRS9, and CPRS15; with scores ranging from 8 to 40), and eight are for closeness (CPRS2, CPRS4, CPRS8, CPRS10, CPRS11, CPRS12, CPRS13, and CPRS14; with scores ranging from 7 to 35). The higher the score one gets on these subscales, the higher the level of conflict or closeness, respectively. The CPRS has shown good psychometric properties in diverse cultures and languages in other studies [44]. In the present study, the short form of the Child–Parent Relationship Scale (CPRS‐SF) was used.

#### 2.1.6 Translation Procedure

Professional translators translated the CPRS‐SF from English to Arabic and then from Arabic to English (forward and then backward translation). This version was compared to the original CPRS‐SF scale, and differences were fixed. Translators did not interact directly with one another, and the process followed the International Test Commission guidelines for test adaptation [45].

### 2.2 Statistical Analysis

Confirmatory factor analysis (CFA) was conducted in R using the lavaan package (version 4.5.2), employing a robust weighted least squares estimator (WLSMV) suitable for ordered categorical data.

A minimum sample size of 300 participants was estimated based on the recommendation of 20 participants per observed variable [46]. Model fit was evaluated using multiple indices: root mean square error of approximation (RMSEA ≤ 0.08), standardized root mean square residual (SRMR ≤ 0.05), Tucker–Lewis index (TLI), and comparative fit index (CFI) (≥0.90 for both) [47]. Because the mediation analysis involved indirect effects, a nonparametric bootstrapping procedure was applied to estimate confidence intervals.

Univariate normality of PMUM scores was assessed using skewness and kurtosis, with values between −1 and +1 considered acceptable [48]. Student's *t*‐test was used to compare two means, ANOVA test for three or more means, and Pearson's correlation to examine associations between continuous variables. Mediation analyses were conducted in R using the lavaan package. A multiple mediation model was specified, including psychological distress (PHQ‐4 total score) and PCC as mediators. Indirect effects were estimated using 5000 bootstrap resamples with percentile‐based confidence intervals. Standardized coefficients are reported. Because the structural mediation model using observed composite scores was just‐identified (df = 0), global model fit indices (e.g., CFI and RMSEA) were not informative; therefore, inference focused on standardized path coefficients, bootstrapped confidence intervals for indirect effects, and explained variance (*R* ^2^). Mediation was considered significant if 95% CI did not include zero. Covariates with *p* < 0.25 in bivariate analyses were included in the model [49]. *p* < 0.05 was considered statistically significant.

#### 2.2.1 Model Specification

The mediation model was estimated using observed composite scores rather than latent variables. PSA, psychological distress (PHQ‐4 total score), PCC (CPRS‐SF conflict subscale score), and child problematic media use (PMUM total score) were entered as observed continuous variables. Covariates (parent age, education level, and child screen time) were included as predictors of the mediators and the outcome to control for potential confounding. Specifically, direct paths were specified from each covariate to psychological distress, PCC, and child problematic media use. Because the mediation analysis relied on composite scale scores, all variables were treated as continuous, and the indirect effects were estimated using bootstrap procedures. The ordered‐categorical treatment of items applied only to the CFA of the CPRS‐SF, which was conducted at the item level using the WLSMV estimator, and did not affect the mediation models based on composite scores.

#### 2.2.2 Missing Data Handling

A total of 892 participants completed the questionnaire. Missing data were minimal across study variables. The proportion of missing values was 1.8% for daily working hours and 0% for all primary variables, including PSA, psychological distress, child–parent relationship dimensions, and child problematic media use. Analyses not involving daily working hours (including CFA, bivariate analyses, and mediation models) were conducted on the full sample of 892 participants. Analyses involving working hours were conducted on complete cases (*N* = 876). Given the very low level of missingness, no imputation procedures were applied.

## 3 Results

### 3.1 Participants

A total of 892 participants completed the questionnaire (mean age = 37.22 years), and 92.7% were married. All characteristics of the sample are presented in Table 1.

**TABLE 1:** Sociodemographic and other characteristics of the sample (*N* = 892).

Variable	*N* (%)
Parent	
No	197 (22.1%)
Yes	695 (77.9%)
Marital status	
Single, divorced, widowed	65 (7.3%)
Married	827 (92.7%)
Education level	
Secondary or less	283 (31.7%)
University	609 (68.3%)
Occupation	
Unemployed	402 (45.1%)
Part‐timer	207 (23.2%)
Full‐timer	283 (31.7%)
Living area	
Urban	504 (56.5%)
Rural	388 (43.5%)
Screen time per day	
Less than 1 h	122 (13.7%)
1–2 h	256 (28.7%)
2–3 h	255 (28.6%)
4 h and above	259 (29%)

	**Mean ± SD**
Age (years)	37.22 ± 8.11
Household overcrowding index (person/room)	1.18 ± 1.75
Working hours per day	4.27 ± 4.68
Smartphone addiction	25.95 ± 10.29
Problematic media use measure (PMUM)	21.52 ± 7.91
Conflict	20.11 ± 7.14
Closeness	25.81 ± 6.93
Psychological distress	4.12 ± 2.91

### 3.2 CFA of the Child–Parent Relationship Scale

CFA supported the original two‐factor structure of the CPRS‐SF, comprising Closeness (seven items) and Conflict (eight items). Using a robust estimator for ordered categorical data, model fit was acceptable, with a scaled CFI of 0.951, a scaled TLI of 0.942, a robust RMSEA of 0.085 (90% CI 0.078, 0.092), and an SRMR of 0.088.

Standardized factor loadings ranged from 0.51 to 0.91 for the Closeness subscale and from 0.71 to 0.79 for the Conflict subscale (Table 2), with all loadings statistically significant (*p* < 0.001). Internal consistency was high for both subscales (Closeness: Cronbach's *α* = 0.91, ω=0.92; Conflict: Cronbach's *α* = 0.88, ω=0.89). Composite reliability (CR) was excellent for Closeness (CR = 0.91) and Conflict (CR = 0.90), and convergent validity was supported by average variance extracted (AVE) values exceeding the recommended threshold of 0.50 (Closeness AVE = 0.68; Conflict AVE = 0.56). The correlation between the two latent factors was small and negative (*r* = −0.09, *p* < 0.001).

**TABLE 2:** Confirmatory factor analysis of the Child–Parent Relationship Scale‐Short Form (CPRS‐SF).

Section A. Standardized factor loadings			
Item	Factor	Standardized loading	SE
CPRS‐SF 1	Closeness	0.888	0.009
CPRS‐SF 3	Closeness	0.506	0.022
CPRS‐SF 5	Closeness	0.889	0.009
CPRS‐SF 6	Closeness	0.914	0.008
CPRS‐SF 7	Closeness	0.874	0.010
CPRS‐SF 9	Closeness	0.815	0.012
CPRS‐SF 15	Closeness	0.821	0.012
CPRS‐SF 2	Conflict	0.733	0.016
CPRS‐SF 4	Conflict	0.710	0.019
CPRS‐SF 8	Conflict	0.739	0.016
CPRS‐SF 10	Conflict	0.741	0.015
CPRS‐SF 11	Conflict	0.741	0.015
CPRS‐SF 12	Conflict	0.748	0.014
CPRS‐SF 13	Conflict	0.788	0.014
CPRS‐SF 14	Conflict	0.785	0.016

**Section B. Reliability and convergent validity**			
**Subscale**	**Cronbach's *α***	**McDonald's** ω	**CR**
Closeness	0.91	0.92	0.91
Conflict	0.88	0.89	0.90
**Section C. Model fit indices**			
Index	Value		
CFI	0.951		
TLI	0.942		
RMSEA	0.085		
90% CI RMSEA	0.078–0.092		
SRMR	0.088		

*Note:* CFA was conducted using a robust estimator for ordered categorical data (WLSMV). All standardized factor loadings were statistically significant (*p* < 0.001). RMSEA values are reported as robust estimates with 90% confidence intervals.

Abbreviations: AVE, average variance extracted; CFA, confirmatory factor analysis; CR, composite reliability; SRMR, standardized root mean square residual; TLI, Tucker–Lewis index.

### 3.3 Measurement Invariance Across Parent Gender

Measurement invariance of the CPRS‐SF across parent gender (fathers vs. mothers) was examined using multigroup CFA with a robust estimator for ordered categorical data. Measurement invariance analyses were conducted to assess the comparability of the CPRS‐SF across parent gender; these analyses were not intended to support causal group comparisons. Configural, metric, scalar, and strict invariance were supported, indicating that the two‐factor structure, factor loadings, item thresholds, and residual variances were comparable across groups (Table 3). Changes in model fit indices between successive models remained within recommended cut‐off values, supporting meaningful comparisons between fathers and mothers. Given evidence of scalar invariance, observed mean comparisons of PCC and closeness scores between fathers and mothers were considered interpretable. Fathers showed higher conflict scores, whereas mothers showed higher closeness scores (Table 4).

**TABLE 3:** Measurement invariance of the Child–Parent Relationship Scale‐Short Form across parent gender.

Model	CFI	RMSEA	SRMR	Model comparison	ΔCFI	ΔRMSEA	ΔSRMR
Configural	0.951	0.093	0.090				
Metric	0.954	0.092	0.091	Configural vs. metric	0.002	−0.002	0.001
Scalar	0.950	0.094	0.090	Metric vs. scalar	−0.003	0.002	−0.001
Strict	0.950	0.094	0.090	Scalar vs. strict	<0.001	<0.001	<0.001

*Note:* Measurement invariance was tested using multigroup CFA with a robust estimator for ordered categorical data (WLSMV). Invariance was evaluated using changes in model fit indices (ΔCFI ≤0.01,ΔRMSEA≤0.015,ΔSRMR≤0.01).

Abbreviations: CFI, comparative fit index; RMSEA, root mean square error of approximation; SRMR, standardized root mean square residual.

**TABLE 4:** Comparison of child–parent relationship subscale scores between parents.

	Father	Mother	*p*	Effect size
Child–parent conflict score	21.20 ± 7.13	19.80 ± 7.12	**0.015**	0.196
Child–parent closeness score	24.82 ± 6.80	26.09 ± 6.95	**0.023**	0.183

*Note:* Numbers in bold indicate significant *p* values.

### 3.4 Preliminary Bivariate Associations With Child Problematic Media Use

At the bivariate level, higher PMUM scores were associated with lower parental education, longer daily screen time, older parental age, higher psychological distress, and greater PCC (Tables 5 and 6). PMUM scores differed significantly across screen time categories, with higher scores observed among parents reporting ≥ 4 h of daily screen exposure.

**TABLE 5:** Association of factors with child problematic media use.

Variable	Mean ± SD	*p*	Effect size (Cohen's *d*)
Parent*		0.165	0.112
No	22.21 ± 7.97		
Yes	21.32 ± 7.88		
Marital status*		0.440	0.099
Single, divorced, widowed	22.25 ± 8.02		
Married	21.46 ± 7.90		
Education level*		**0.007**	0.193
Secondary or less	22.55 ± 7.93		
University	21.03 ± 7.85		
Occupation**		0.315	0.893
Unemployed	21.76 ± 7.85		
Part‐timer	21.86 ± 8.38		
Full‐timer	20.93 ± 7.62		
Living area*		0.368	0.061
Urban	21.73 ± 7.93		
Rural	21.24 ± 7.88		
Screen time per day**		**<0.001**	4.862
Less than 1 h	18.83 ± 8.19		
1–2 h	18.68 ± 7.73		
2–3 h	22.09 ± 7.19		
4 h and above	25.03 ± 7.09		

*Note:* Numbers in bold indicate significant *p* values. *Statistical test = Student t; **Statistical test = ANOVA. The Bonferroni post hoc analysis showed no difference between groups for the occupation variable. However, for the screen time variable, a significant difference was found between two categories (*p* < 0.001), except for less than 1 h versus 1–2 h, which showed no significant difference (*p* = 1).

**TABLE 6:** Pearson correlation matrix.

	1	2	3	4	5	6
1. Child's problematic media use	1					
2. Age	0.09^**^	1				
3. Household overcrowding index	0.01	0.08^*^	1			
4. Working hours per day	0.03	0.10^**^	0.02	1		
5. Parental psychological distress	0.29^***^	0.01	−0.01	0.04	1	
6. Child–parent conflict	0.45^***^	0.05	0.03	0.02	0.28^***^	1
7. Child–parent closeness	−0.05	−0.03	0.01	−0.02	−0.19^***^	−0.01

* *p* < 0.05.

** *p* < 0.01.

*** *p* < 0.001.

These analyses are descriptive and unadjusted and were conducted to explore crude associations and to guide the selection of covariates for subsequent multivariable mediation analyses.

Variables showing associations at *p* < 0.25 in bivariate analyses were considered for inclusion as covariates in the multivariable mediation models, in line with recommended model‐building strategies.

### 3.5 Mediation Analyses

**FIGURE 2:**
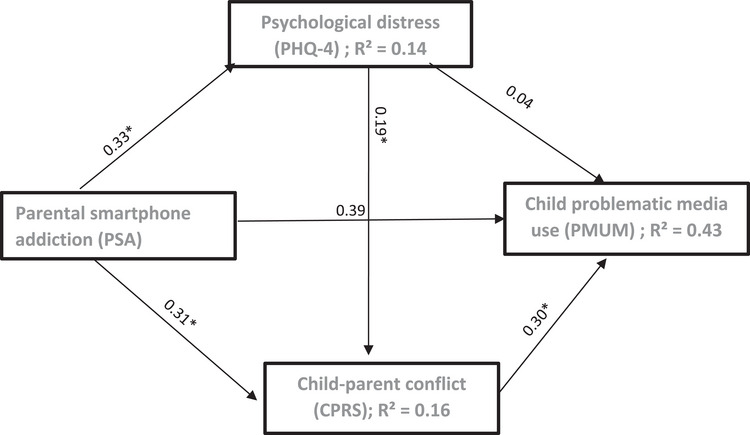
Structural equation model illustrating the multiple mediation of the association between parental smartphone addiction and child problematic media use. Standardized coefficients are displayed. The model was adjusted for parent age, education level, and child screen time.

A multiple mediation model was tested to examine whether psychological distress (PHQ‐4 total score) and PCC mediated the association between PSA and child problematic media use. Indirect effects were estimated using 5000 bootstrap resamples, adjusting for parent age, education level, and child screen time. The mediation was estimated within a structural equation modeling (SEM) framework; because the structural mediation model was just identified (df = 0), global model fit indices were not informative, and interpretation focused on standardized path coefficients, bootstrapped confidence intervals, and explained variance (Figure 2).

The indirect effect through psychological distress alone was not significant. In contrast, PCC significantly mediated the association between PSA and child problematic media use. A significant serial indirect effect was also observed, whereby higher PSA was associated with greater psychological distress, which, in turn, predicted higher PCC and increased child problematic media use. The total indirect effect was significant, whereas the direct association between PSA and child problematic media use remained significant after accounting for the mediators (Table 7).

**TABLE 7:** Direct, indirect, and total effects of parental smartphone addiction on child problematic media use.

Effect	Path	Standardized *β*	95% bootstrap CI	*p* value
Direct effect	PSA ∈ PMUM	0.30	[0.26, 0.34]	<0.001
Indirect (PHQ‐4 only)	PSA ∈ Psychological distress ∈ PMUM	0.01	[−0.01, 0.03]	—
Indirect (Conflict only)	PSA ∈ Conflict ∈ PMUM	0.09	[0.06, 0.12]	—
Serial indirect	PSA ∈ Psychological distress ∈ Conflict ∈ PMUM	0.02	[0.01, 0.03]	—
Total indirect	—	0.12	[0.08, 0.16]	—
Total effect	PSA ∈ PMUM	0.39	[0.35, 0.43]	<0.001

*Note:* Estimates are standardized coefficients. Confidence intervals were obtained using 5000 bootstrap resamples. Models adjusted for age, education level, and child screen time. Significant effects are those whose 95% CI does not include zero.

Abbreviations: PMUM, problematic media use measure; PSA, parental smartphone addiction.

The mediation model explained a substantial proportion of variance in child problematic media use (*R* ^2^ = 0.43). The mediators accounted for 16% of the variance in PCC and 14% of the variance in parental psychological distress, indicating moderate explanatory power of the proposed pathways.

## 4 Discussion

Our mediation analysis examined how PSA relates to child problematic media use within the Lebanese context, guided by the IT‐CPU. The findings provide strong support for the theory's proposition that distal parental factors (like PSA) are associated with child outcomes through more proximal and maintaining factors, specifically parental mental health and the parent–child relationship. These findings are consistent with the IT‐CPU, which posits that parental characteristics and family relational processes jointly shape children's media behaviors [21].

### 4.1 Validation of CPRS‐SF in Arabic

The psychometric qualities obtained from the validation of the CPRS‐SF in Arabic align with results from studies conducted in Turkey, the United States, the United Kingdom, and Italy [33, 34, 50, 51]. Beyond demonstrating equivalence with prior validations, the present study extends the literature by providing structural validity evidence for the CPRS‐SF in an understudied Arab cultural context, where parenting norms and relational expectations may differ from Western settings in which the scale was originally developed.

The two‐factor structure—emphasizing conflict and closeness—derived from the original CPRS‐SF was supported by our CFA and showed good model fit. These results are consistent with the Italian validation by Rinaldi et al., who reported acceptable fit indices [44]. However, unlike Rinaldi et al., who supported a three‐factor model (conflict, closeness, and dependency), our findings retained the original two‐factor structure. This suggests that dependency may not emerge as a distinct relational dimension in Arabic‐speaking samples, potentially reflecting cultural differences in conceptualizations of child autonomy and parental involvement. The divergence between models may also be attributable to methodological choices, as Rinaldi et al. removed items that participants found difficult to understand. The long form of the CPRS was previously successfully validated in Turkish samples [52, 53]. Although the SRMR slightly exceeds the conservative cutoff of 0.05 [47], it remains below the more commonly accepted threshold of 0.08 [54]. Therefore, model fit should be interpreted holistically rather than through strict cutoffs, especially in cross‐cultural studies. Taken together with the remaining fit indices, the model is reasonably adequate.

Interestingly, the correlation between the Closeness and Conflict subscales was very low (*r* = −0.05), showing slight overlap. Rather than indicating a measurement problem, this may reflect cultural dynamics in which emotional closeness and conflict coexist in Lebanese and other collectivist families without being perceived as contradictory. This pattern aligns with cultural norms where parental authority and emotional involvement exist together rather than erase each other. Translation differences or sampling characteristics (e.g., relying on self‐reported data from a convenience sample) may also have contributed to this low correlation. Future multi‐informant designs are needed to clarify whether this independence reflects cultural reality or methodological constraints.

Internal consistency was strong (*α* = 0.88 for conflict and 0.91 for closeness) and comparable to or higher than previous validations (*α* = 0.80 and 0.75 in parents of 2‐year‐olds in the United Kingdom [33] and 0.69 for both subscales in Turkey [34]). These findings further support that our Arabic CPRS‐SF is more reliable than validations in other cultural contexts. Additionally, measurement invariance across gender was established, supporting meaningful comparisons between mothers and fathers. This makes an important contribution, as few studies in Arab contexts have tested gender invariance in the parent–child relationship measures [55].

Consistent with prior research, mothers reported higher closeness scores than fathers, whereas fathers reported higher conflict scores. These differences reflect culturally assigned parenting roles, where mothers are more involved in emotional caregiving and fathers are more associated with discipline and setting boundaries [56]. Lebanon represents a sociocultural context in which Western and traditional Arab values coexist, reflected in the nearly equal prevalence of authoritative (31.4%) and authoritarian (29.3%) parenting styles [36]. Within this context, the concept of “patriarchal connectivity,” which describes the coexistence of family interdependence, emotional closeness, and parental authority in collectivist societies, helps contextualize our findings [57]. This framework is used here as a contextual lens to interpret findings rather than as a direct causal explanation. From this perspective, excessive smartphone use by parents or children may be perceived as emotional detachment from the family unit. PSA may be interpreted as reduced availability, whereas children's prolonged smartphone use may be interpreted as detachment from shared family identity, potentially increasing relational tension [58].

#### 4.1.1 Mediation Findings

Our findings extend existing research by specifying how PSA contributes to child problematic media use through two indirect pathways: parental psychological distress and PCC. First, parental psychological distress did not function as a significant indirect pathway linking PSA to child problematic media use. This suggests that PSA may not undermine parental psychological distress symptoms, reducing their emotional availability, consistency, and self‐regulation [1]. In turn, children may rely on media as a coping strategy for emotional neglect or distress [59]. Importantly, this pathway highlights that PSA‐related risks are not limited to modeling excessive media use but are also associated with problems that affect parents’ psychological functioning [60].

Second, PCC demonstrated a stronger indirect effect than psychological distress, highlighting its central role within the IT‐CPU framework. Rather than monitoring alone, relational strain appears to be a key mechanism linking PSA to child problematic media use. Excessive PSA may reduce interaction quality and increase irritability, inconsistency, and misunderstanding, leading to maladaptive media use in children [61, 62]. This pattern aligns with negativity bias theory, which suggests that negative relational experiences exert stronger behavioral effects than positive ones [63]. Crucially, this pattern must be interpreted within the Lebanese and broader Arab sociocultural context, which is characterized by collectivist values that emphasize family cohesion, hierarchy, and interdependence [64]. In these cultures, closeness between parents and children is the normal baseline rather than an active protective factor. Consequently, variations in closeness do not have enough explanatory power. Moreover, in collectivist Arab families, parental restriction and control, especially regarding use of technology, are usually perceived by children as expressions of care rather than intrusion [65].

Finally, these findings reflect transactional dynamics between parents and their children. Although PSA contributes to parental psychological distress and conflict, children's capacity at emotional regulation may also affect family dynamics. Children who struggle with regulation might respond more intensely if parents are unavailable, which escalates the conflict and reinforces their own problematic media use [5, 7]. This bidirectional view emphasizes how complex the family processes related to PSA are and warns against simple linear interpretations. In summary, this study contributes to literature by not only describing associations but also identifying specific relational and psychological mechanisms. It also demonstrates how the cultural context of Lebanese and Arab families shapes these mechanisms. These insights encourage culturally sensitive interventions that could target parental well‐being and conflict management, rather than only focusing on reducing children's screen time.

## 5 Theoretical and Clinical Implications

This study helps better understand the IT‐CPU by clarifying how “proximal” (parental psychological distress) and “maintaining” (parental child conflict) factors operate in Lebanese families. These results suggest that culturally informed adaptations of the IT‐CPU model may be warranted, particularly in collectivist contexts where family hierarchy and interdependence shape relational meanings. Additionally, the validation of the Arabic version of the CPRS provides researchers, clinicians, and school professionals with a culturally appropriate instrument to assess relational dynamics across Arab countries. This will enable early identification efforts in schools and healthcare settings.

From a clinical and public health perspective, the findings highlight the importance of preventive approaches that are family‐centered. These should target the identifying mediating mechanisms rather than only focusing on children's media use. Routine screening for PSA and psychological distress symptoms during pediatric or school health visits may help interrupt the intergenerational cycle leading to child problematic media use.

Finally, intervention efforts should prioritize improving parent–child communication, emotional regulation, and conflict resolution. Evidence‐informed resources, such as Common‐Sense Media's Family Engagement Framework [66], the World Health Organization's “Doing What Matters in Times of Stress: An Illustrated Guide” [67], and family‐based cognitive behavioral interventions, offer practical, scalable strategies to reduce family conflict, enhance parental responsiveness, and support children's self‐regulation. Implementing such interventions may mitigate the negative impact of PSA on children's media behaviors and overall psychosocial development.

## 6 Limitations

Although mediation analyses were conducted to examine theoretical pathways, the cross‐sectional design precludes causal inference. The observed indirect effects should therefore be interpreted as statistical associations consistent with the proposed model rather than evidence of temporal or causal mechanisms. Alternative explanations, including reverse or bidirectional associations (e.g., child problematic media use contributing to parental stress and smartphone use), cannot be ruled out. Additionally, to the use of respondent‐completed surveys, we expect to encounter information bias due to possible incorrect answers or due to misunderstanding questions or social desirability bias. Furthermore, the snowball sampling technique reduces the representativeness of the sample and may have introduced selection bias as participants were recruited through networks of similar socioeconomic and educational backgrounds. Future studies should use probabilistic or stratified sampling methods to enhance generalizability. Given the use of self‐reported data from a cross‐section study, the potential for common method variance (CMV) was considered, which might lead to inflation of the associations among variables due to shared method rather than actual underlying effects. We tried to lower the CMV by using validated scales and ensuring anonymity, although the risk of CMV cannot be completely ruled out. In addition, the mediation model showed a differentiated pattern of effects, with the indirect pathway through psychological distress alone being nonsignificant, whereas the conflict and serial pathways were significant. If the results were driven purely by shared‐method variance, similar inflation would be expected across all pathways. This pattern therefore suggests that the observed associations are unlikely to be solely attributable to common method bias. Nevertheless, future research should replicate these findings using multi‐informant designs, including child‐reported media use and observational or teacher‐reported measures of the parent–child relationship, as well as longitudinal data to better establish temporal ordering. Another limitation in this study was the reliance on parental self‐reports without getting the perspective of children on PCC or media use, which could give a partial and distorted representation of the dynamics. Further research should use multi‐informant designs, asking both parents and children for a better understanding. Finally, although we adopted the International Test Commission guidelines for test adaptation for the Arabic translation of the CPRS‐SF, additional psychometric properties (e.g., test–retest) and validation across many Arabic‐speaking populations are still required [68]. Despite these limitations related to the design and sampling, our study offers valuable information about smartphone addiction patterns and family dynamic in the Lebanese context. These results are a solid foundation for future longitudinal research as well as creating targeted interventions in the Middle East.

## 7 Conclusion

In conclusion, this study supports a complex relationship between PSA and child problematic media use in the Lebanese context. It also successfully validates the Arabic version of the CPRS‐SF. Parental psychological distress and PCC emerged as important mediators emphasizing the interconnection between family dynamics and technology‐related behaviors. Longitudinal studies are needed to clarify temporal and bidirectional dynamics. Furthermore, clinical trials should test culturally adapted family‐based interventions. Culturally sensitive prevention strategies targeting parental well‐being and relational functioning may be more effective than child‐focused screen reduction approaches alone.

## Author Contributions

Sahar Obeid, Feten Fekih‐Romdhane, and Souheil Hallit designed the study; Nicole Tannous, Marilyne Aouad, and Abdallah Chahine drafted the manuscript; Souheil Hallit carried out the analysis and interpreted the results; Fouad Sakr and Mariam Dabbous collected the data. Diana Malaeb, Rabih Hallit and Sami El Khatib and all authors reviewed the article for intellectual content and gave their consent.

## Funding

The authors have nothing to report.

## Ethics Statement

The Lebanese International University's School of Pharmacy ethics committee granted this study ethics permission (IRB 2024ERC‐045‐LIUSOP). When filling out the online form, parents provided written informed consent. Participation was voluntary. Every step of the process was carried out in compliance with all applicable laws and rules (such as the Declaration of Helsinki). Confidentiality and anonymity were maintained as no identifying information (such as names, email addresses, or IP addresses) was collected. Data were stored securely and used only for research purposes.

## Consent

The authors have nothing to report.

## Conflicts of Interest

The authors declare no conflicts of interest.

## Data Availability

Because of ethical committee constraints, none of the data collected or analyzed during this study are publicly available. However, the corresponding author (Souheil Hallit) may make the data available upon reasonable request.
